# Advances and Emerging Therapies in the Treatment of Non-alcoholic Steatohepatitis

**DOI:** 10.17925/EE.2022.18.2.148

**Published:** 2022-11-22

**Authors:** Paul N Brennan, John F Dillon, Rory McCrimmon

**Affiliations:** 1. The University of Edinburgh, Centre for Regenerative Medicine, Edinburgh, UK; 2. NHS Tayside, Ninewells Hospital and Medical School, Dundee, UK; 3. The University of Dundee, Department of Molecular and Clinical Medicine, Ninewells Hospital and Medical School, Dundee, UK

**Keywords:** Fatty liver disease, fibrosis, NAFLD, NASH, therapeutics

## Abstract

Non-alcoholic steatohepatitis (NASH) now represents one of the most prevalent forms of cirrhosis and hepatocellular carcinoma. A number of treatment agents have undergone assessment in humans following promising results in animal models. Currently, about 50 therapeutic agents are in various stages of development. Recently, however, there have been a number of exciting and positive developments in this landscape, although there are inherent challenges ahead. In this article, we review the aetiological and pathological basis of NASH progression and describe putative targets for current therapies. We also discuss some of the likely future directions and difficulties around this complex and challenging disease paradigm.

Non-alcoholic fatty liver disease (NAFLD), which impacts an estimated 25% of the world’s adult population,^1–3^ is the principal cause of chronic liver disease globally. NAFLD as a whole represents a pathological spectrum of liver injury, spanning from simple steatosis to non-alcoholic steatohepatitis (NASH) and liver fibrosis, with an evolutionary course to cirrhosis and risk of hepatocellular carcinoma. Within the continuum, NAFLD develops into NASH in 20% of cases,^2^ with NASH being a leading cause of further progression to liver cirrhosis and cancer,^4^ and NASH-related cancer being the second major cause of years of life lost among all cancers.

The association between NAFLD development and obesity, insulin resistance and type 2 diabetes mellitus (T2DM), is well established.^5^ Given the increasing prevalence of these related conditions, the incidence of NAFLD is projected to increase, with data suggesting a 56% rise over the next decade.^2^ Although NAFLD is typically associated with a Western lifestyle, data demonstrate a rapid increase in disease burden in developing counties.

The pathogenesis of NAFLD is complex and thought to be dependent on ‘multiple parallel hits’ on a background of genetic susceptibility. NAFLD progression is best considered as a dynamic two-way process relating to repetitive bouts of metabolic stress and inflammation, interspersed with endogenous anti-inflammatory reparative responses. Recent advances in deciphering the pathogenesis of NAFLD, including predisposing genetic determinants (*PNPLA3*, *TM6SF2*, *HSD17b*, *MOAT7*)^6^ and identification and validation of involved biomarkers,^7^ have improved our understanding of this disease, as well as allowing the development of tools to stratify disease severity and prognosis.^8,9^ However, there remain significant knowledge gaps relating to susceptibility and progression variability between individuals.

Despite numerous clinical trials, there are no licensed pharmacological interventions for NAFLD. In the absence of approved drug treatments, lifestyle interventions remain pivotal in the management of NAFLD across its entire disease continuum. There is a strong correlation between weight loss and the resolution of NAFLD, including fibrosis regression, and therefore therapies that induce weight loss are obviously an attractive drug target.^10^

Given emerging insights in NAFLD pathogenesis, it is possible that multiple tangential pathways are engaged to successfully alter the natural history of the disease.^11^ Although there are currently no licensed therapeutics for NAFLD, there are pharmacological agents for other components of the metabolic syndrome. Despite biological plausibility, and some preliminary suggestions around efficacy, none of these have unequivocally achieved prerequisite endpoints.^12^ The optimal combination of these therapies is again likely subject to various metabolic, genetic and geneenvironment considerations.

**Table 1:** Overview of putative molecular targets and specific agents trialled in non-alcoholic steatohepatitis

Molecular target	Pharmaceutical agent
Peroxisome proliferator-activated receptor (PPAR) agonists (receptor specificity)	Bezafibrate (α) Fenofibrate (α) Pioglitazone (γ) Rosiglitazone (γ) Saroglitazar (α/γ) Elafibranor (α/δ) Lanifibranor (α/δ/γ)
Thyroid hormone receptor β (THR-β)	Resmetirom
Fibroblast growth factor (FGF) (subclass)	Aldafermin (FGF-19 analogue) Pegozafermin (FGF-21 analogue) Pegbelfermin (FGF-21 analogue)
Glucagon-like peptide 1 (GLP-1) agonists	Liraglutide Semaglutide
Farnesoid X receptor (FXR) agonists	Obeticholic acid (OCA)
Chemokine receptor (CCR) antagonist (receptor specificity)	Cenicriviroc (CCR-2, CCR-5 dual antagonist)
Metabolic enzyme inhibitors (specific enzyme)	Firsocostat (ACC inhibitor) PF-05221304 (ACC inhibitor) PF-06865571 (DGAT-2) Aramchol (SCD-1 inhibitor)

*ACC = acetyl-CoA carboxylase; DGAT-2 = diacylglycerol acyltransferase 2;*

*SCD-1 = stearoyl-CoA desaturase-1.*

An interesting novel approach to some of the challenges within the area relate to the plausibility of the variability in liver homeostasis as influenced by the circadian clock. This evolutionarily conserved physiological mechanism controls highly coordinated aspects of metabolism including fatty acid synthesis, signalling of farnesoid X receptor (FXR), fibroblast growth factor (FGF) 19 and 21, peroxisome proliferator-activated receptor (PPAR) α and γ, glucagon-like peptide 1 (GLP-1) and thyroid hormone receptor (THR).^13^ These circadian rhythms have significant implications for targeted dosing regimens as part of potential clinical trials. The evidence for this is too extensive for the purpose of this review; however, we would direct readers to an excellent review on the topic by Marjot et al.^13^

In this review, we highlight some of the novel therapeutic targets for NASH currently undergoing clinical trials. A brief outline of these targets and associated compounds are summarized in *Table 1*, and an overview of the most important pathways is provided in *Figure 1*.^14^

## Peroxisome proliferator-activated receptors

Peroxisomes are intrinsically implicated in normal fatty acid catabolism, in addition to contributing to normal energy metabolism via the pentose phosphate pathway.^15^ PPAR signalling characteristically involves multiple cellular organelles, including mitochondria, with pleiotropic effects, thereby influencing glucose metabolism, inflammatory processes and fibrogenesis.^16^ Three distinct PPAR isotypes have been well characterized, α, β/δ and γ, which exhibit differential expression and actions depending on isotype, organ and intra-organ cell type.^16^

Pioglitazone (with vitamin E) has historically demonstrated histological improvements in NASH across a number of randomized controlled trials (RCTs), although it has not received US Food and Drug Administration approval as a licenced treatment.^17,18^ It is, however, licensed as a treatment for T2DM. Therefore, it can be used for persons with co-existent T2DM and NAFLD. Pioglitazone is effective in improving glucose homeostasis, and mobilizes visceral adipose tissue, further influencing its glucose-lowering potential. Similarly, pioglitazone has been shown to have potent modulatory effects in reducing inflammation in coronary vessels.^19^

### Lanifibranor

As suggested, PPARs are nuclear receptors with an array of diverse regulatory functions including metabolic and inflammatory coordination, and regulation of fibrogenesis.^20^ In preclinical models, the indole-sulfonamide derivative, lanifibranor (IVA337), a pan-PPAR agonist, improved insulin sensitivity and macrophage activation, with consequent reduction in liver fibrosis and inflammatory gene expression with higher efficacy than single or dual PPAR agonists.^20,21^

Lanifibranor was evaluated in a phase IIb, double-blind RCT in patients with non-cirrhotic and highly active biopsy-confirmed NASH (NATIVE study).^22^ Randomization occurred in a 1:1:1 ratio, whereby patients received placebo, lanifibranor 800 mg or lanifibranor 1,200 mg, once daily for 24 weeks. T2DM, a strong determinant in NASH pathogenesis, was a stratification factor applied to balance the assignment of patients to the three arms. The NATIVE study design, rationale and outline have been described previously.^23^ The statistical plan hypothesized that the rate of response would be 10% in the placebo group and an excess rate of 20% for any dose of investigational medicinal product, thereby necessitating 72 patients per arm.

The primary endpoint was a reduction of at least 2 points in component A of the Steatosis, Activity, Fibrosis scoring system.^22^ Exploratory secondary endpoints included regression of fibrosis or resolution of NASH. In total, 247 patients were randomized, with 188 (76%) having moderate to advanced fibrosis. Overall, 55% of those allocated to lanifibranor 1,200 mg met the primary endpoint versus 33% in the placebo group (p=0.007); however, the 800 mg dose did not achieve statistical significance compared with placebo (48% versus 33%; p=0.07). Results also favoured the 1,200 mg and 800 mg doses over placebo in achieving improvement in fibrosis stage of at least 1 without worsening of NASH (48% and 34% respectively, versus 9% in placebo). Similarly, there was associated improvement in liver enzymes and lipid, inflammation and fibrosis biomarkers in the treatment cohorts.

Clearly, PPAR modulation represents a promising target in NASH, given the relative success of PPAR-γ effects noted from the PIVENS trial and other longitudinal datasets,^18^ with the suggestion that pan-PPAR agonism is likely to demonstrate true clinical benefit across all major accepted primary and secondary endpoints. Importantly, stratification according to T2DM was included during allocation to ensure patients were representative across all cohorts.

## Thyroid hormone receptor β

There is evolving evidence to suggest that NASH may in part be a consequence of diminished liver thyroid hormone levels or as a variant of functional hepatic hypothyroidism. This theory has been extrapolated from studies that demonstrated a higher incidence of hypothyroidism in patients with NAFLD/NASH relative to population age- and sex-matched controls,^24^ in addition to a putative molecular pathway.^25^

In NASH, selectivity for THR-β may provide metabolic benefits of thyroid hormone mediated by the liver, including modulating hepatic steatosis, reducing atherogenic lipids (low-density lipoprotein–cholesterol, triglycerides), and lipoproteins (apolipoprotein B and CIII, lipoprotein[a]), while minimizing systemic sequelae related to excess exogenous thyroid hormone administration, particularly relating to cardiac and bone effects, which are principally mediated via THR-α.^24^

**Figure 1:**
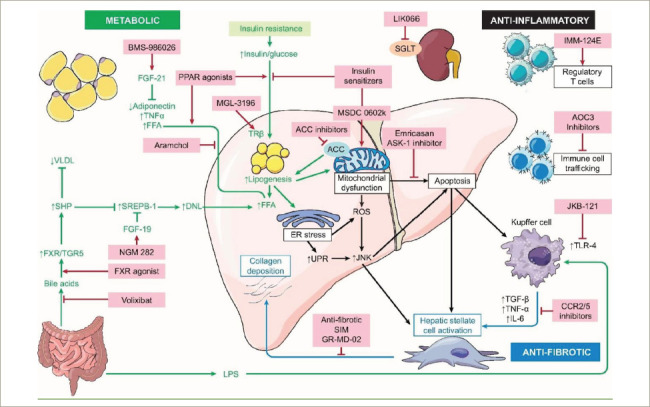
Overview of putative pathways implicated in non-alcoholic steatohepatitis pathogenesis and molecular targets

*From Konerman MA et al.^14^*

*ACC = acetyl-CoA carboxylase; AOC = amine oxidase copper-containing 3; ASK = apoptosis signal regulating kinase; CCR = chemokine receptor; DNL = de novo lipogenesis; ER = endoplasmic reticulum; FFA = free fatty acid; FGF = fibroblast growth factor; FXR = farnesoid X receptor; IL = interleukin; JKB = JKB121 (TLR4 antagonist); JNK = c-Jun N-terminal kinase; LPS = lipopolysaccharide; PPAR = peroxisome proliferator-activated receptor; ROS = reactive oxygen species; SGLT = sodium glucose cotransporter; SHP = Src homology 2 domain containing protein tyrosine phosphatase; SIM = simtuzumab; SREPB = sterol regulatory element binding protein; TGF = transforming growth factor; TLR = toll-like receptor; TNF = tumour necrosis factor; TR*β *= thyroid Hormone-*β*; UPR = unfolded protein response; VLDL = very-low density lipoprotein.*

### Resmetirom

Resmetirom (MGL-3196) acts as a selective THR-β agonist, which demonstrates a 28-fold higher affinity than triiodothyronine for THR-β than the co-expressed THR-α receptor.^26,27^ It is inherently liver specific, being highly protein bound (99%) and has poor tissue penetration outside of hepatic parenchyma.^28^

A double-blind RCT study of 84 patients (and 41 controls) with biopsyconfirmed NASH using resmetirom for 36 weeks was undertaken across 25 sites in the USA.^29^ Within this trial, patients had a presumptive diagnosis suggestive of NASH, based on the presence of the metabolic syndrome, plus vibration-controlled transient elastography consistent with liver fibrosis, and steatosis based on a controlled attenuation parameter, or metabolic syndrome plus a previous liver biopsy consistent with NASH with non-cirrhotic fibrosis.^29^ Additionally, patients were required to have a minimum of 10% hepatic fat on screening magnetic resonance imaging proton density fat fraction (MRI-PDFF), before being eligible for liver biopsy to confirm prerequisite criteria for enrolment. Biopsy criteria included evidence of stage 1–3 fibrosis, with NAFLD Activity Score (NAS) score of ≥4, including fulfilment of each component of the score (i.e. ≥1 of each: steatosis, balloon degeneration and lobular inflammation).

Patients were assigned on a randomized basis of 2:1 by a computerbased system to receive resmetirom 80 mg, or matched placebo, orally once daily. Serial hepatic fat measurements were obtained at weeks 12 and 36, and a second liver biopsy was obtained at week 36. The primary endpoint was relative change in MRI-PDFF-determined hepatic fat compared with placebo at week 12 in patients who underwent baseline and week 12 MRI-PDFF.

Resmetirom-treated patients (n=78) demonstrated relative reduction of hepatic fat compared with placebo (n=38) at week 12 (-32.9% resmetirom versus -10.4% placebo; p<0.0001) and week 36 (-37.3% resmetirom [n=74] versus -8.5 placebo [n=34]; p<0.0001). Reported adverse events were predominantly mild or moderate, and were equally distributed across both groups. A phase III, 52-week, open-label, active treatment extension study to evaluate safety and tolerability of once-daily administration of resmetirom (MGL-3196) is ongoing (MAESTRO-NAFLD-OLE, ClinicalTrials. gov: NCT04951219)^30^ in parallel with the double-blind RCT of resmetirom in approximately 2,000 patients using 80 mg or 100 mg daily versus placebo (MAESTRO-NAFLD-1, ClinicalTrials.gov: NCT04197479).^31^

These results highlight the clear potential of THR-β modulation using resmetirom, which on the whole was remarkably well tolerated. Available topline data suggest that MAESTRO-NAFLD has achieved requisite endpoints, and that the last component of the trial series, MAESTRO-NASH,^32^ which contains serial histological assessments, should read-out shortly. The method in which resmetirom has undergone assessment is to be applauded; through a series of parallel, composite trials, investigators were able to maximize recruitment potential, explore variation across both NAFLD and NASH, and minimize variability in drug allocation among cohorts. It is possible that resmetirom may be the first licensed treatment for individuals with NASH.

## Fibroblast growth factor

The FGF-19 subfamily, comprises FGF-19, FGF-21 and FGF-23. FGF-21 is predominantly secreted by the liver, with a broad continuum of tissuespecific autocrine-, paracrine-, and endocrine-mediated metabolic pathways.^33^ Of note, FGF-21 induces production and secretion of adiponectin through PPAR-γ in adipose tissue and is capable of inducing peroxisome proliferator-activated receptor gamma coactivator 1α (PGC-1α).^34^ Circulating levels of FGF-21 and FGF-21 mRNA expression are increased in individuals with NAFLD.

### Pegbelfermin

Pegbelfermin (BMS-986036), a PEGylated human FGF-21 analogue, which previously improved markers of liver fibrosis in obese patients with T2DM, was the subject of a phase II double-blind, randomized clinical trial.^35^ Patients with NASH with fibrosis staging 1–3 were allocated to pegbelfermin 10 mg once daily (n=25), pegbelfermin 20 mg once weekly (n=24) or placebo (n=26), stratified in 1:1:1 ratio and adjusted for diabetes status.

Within the trial there was a significant improvement in absolute hepatic fat fraction in both treatment groups (pegbelfermin 10 mg [daily] versus placebo: -6.8% versus 1.3%, p=0.0004; pegbelfermin 20 mg [weekly] versus placebo: -5.2% versus -1.3%, p=0.008). The trial did not assess histological changes at the end of treatment. A further phase IIb doubleblind RCT evaluating the safety and efficacy of BMS-986036 (PEG-FGF21) in adults with NASH and stage 3 liver fibrosis (FALCON1, ClinicalTrials. gov Identifier: NCT03486899) is currently in active follow-up and should report in the near future.^36^

### Pegozafermin

Results from a phase Ib/IIa proof-of-concept study evaluating pegozafermin (formerly BIO89-100) for the treatment of NASH have recently been reported (ENLIVEN, Clinicaltrial.gov Identifier: NCT04929483).^37^ This study included a single-arm cohort of patients with biopsy-confirmed fibrosis stage F2 and F3 who were treated with pegozafermin 27 mg once weekly for 20 weeks. Approximately 65% of patients at baseline had stage F3 fibrosis. The cohort comprised 20 patients, and 19 received an end-of-treatment biopsy to allow assessment of histology and non-invasive biomarkers.

The primary endpoint was 2-point or greater improvement in NAS without worsening of fibrosis, and was achieved in 63%, with 47% having NASH resolution or fibrosis improvement. Exploratory outcomes of non-invasive tests included MRI-PDFF (-64% mean change from baseline), FibroScan-aspartate aminotransferase score (-76% mean change from baseline) and transient elastography of -31% mean change from index assessment.

Clearly, there are inherent biases within a single cohort study; however, there is a clear suggestion that pegozafermin has potential efficacy, with further RCTs necessary. ENLIVEN is a phase II double-blind RCT that aims to evaluate the efficacy, safety and tolerability of BIO89-100 in a cohort of 216 patients with NASH and is expected to complete in 2023. The primary endpoints will include histological resolution of NASH without worsening of fibrosis and ≥1 stage decrease in fibrosis with no worsening of NASH staging.

### Aldafermin

Aldafermin is an analogue of FGF-19, which acts through inhibition of bile acid synthesis and regulates metabolic homeostasis. Recently, Harrison et al.^38^ reported results from a 24-week, phase II study, which utilized serial liver biopsies as an outcome in patients with NASH.

Within this trial, 78 patients with an NAS score ≥4, stage 2 or 3 fibrosis by NASH Clinical Research Network (CRN) classification, and absolute liver fat content >8% were recruited. Patients were allocated in a 2:1 ratio to aldafermin 1 mg once daily (n=53) or placebo (n=25) for 24 weeks.^38^

The primary outcome was absolute improvement in liver fat content from index scan to that achieved at week 24. Exploratory secondary outcomes examined serum biomarkers and specific histological measures of fibrosis improvement, including NASH resolution. At conclusion of the trial, the aldafermin group met the primary endpoint (7.7% fat reduction compared with 2.7% in the placebo group; p=0.002), with significant changes noted in other biochemical markers including 7α-hydroxy-4-cholesten-3-one, bile acids, aminotransferases and neoepitope-specific N-terminal pro-peptide of type III collagen (PRO-C3) in the treatment cohort. Histological improvements were less impressive, with fibrosis improvement of ≥1 stage with no worsening of fibrosis achieved in 38% of those receiving aldafermin versus 18% of the placebo group (p=0.10). Similarly, NASH resolution failed to achieve significance. A similarly designed phase IIb/III study (ALPINE), recruited 171 patients, and examined additional dose scheduling (0.3 mg, 1 mg and 3 mg) compared with placebo. The primary endpoint was again improvement of liver fibrosis by ≥1 stage with no worsening of NASH at 24 weeks. Again, unfortunately, this endpoint was not achieved according to a topline data release, although full processing of the results is awaited.^39,40^ Again, it appears that multiple secondary endpoints were achieved in the treatment group, including reduction in hepatic steatosis as measured by MRI-PDFF, transaminase and PRO-C3 levels.

These results demonstrate the potent mechanism of the FGF-19 and -21 analogues in reducing hepatic steatosis. However, this hepatic fat reduction clearly does not uniformly offset the other pathogenic elements potentiating NASH, and aldafermin failed to demonstrate the requisite resolution endpoints on histological assessment. Aldafermin is unlikely to be pursued again as a potential strategy, whereas the FGF-21 analogues pegbelfermin and pegozafermin may yet yield positive outcomes.

## Glucagon-like peptide 1 agonists

GLP-1 agonists were originally licenced for treatment in T2DM. Hepatocytes lack GLP-1 receptor expression^41^ and therefore the potential mechanisms through which GLP-1 agonists exert an effect in NASH are likely to relate to improvements in weight and insulin resistance, coupled with reductions in mitochondrial dysfunction, proinflammatory mediators and lipotoxicity.^41–43^

### Semaglutide

Investigation of semaglutide aimed to build on the encouraging early signs noted within the LEAN trial of liraglutide - another GLP-1 agonist (see below).^44^ A 72-week, double-blind, placebo-controlled, phase II trial with biopsy-confirmed NASH was undertaken by Newsome et al.^45^ Within the trial cohort were histological grades F1–F3, with those randomized to treatment receiving 0.1, 0.2 or 0.4 mg of subcutaneous semaglutide or placebo.

The primary endpoint was the resolution of NASH with no progression of fibrosis, and secondary endpoints included improvement of fibrosis staging with no increased histological NASH activity. The secondary endpoint relating to regression of fibrosis was limited to those with F2/ F3 disease accordingly.^45^

A total of 320 patients (230 with F2 or F3 fibrosis) were randomized to receive semaglutide 0.1 mg (n=80), 0.2 mg (n=78), 0.4 mg (n=82) or placebo (n=80). NASH resolution was achieved with no worsening of fibrosis in 40% (0.1 mg), 36% (0.2 mg) and 59% (0.4 mg) of those treated with semaglutide, compared with 17% in the placebo group (p<0.001 semaglutide 0.4 mg versus placebo). In terms of achieving improvement in overall histological fibrosis, there was no appreciable difference between groups, or between those receiving treatment or placebo (0.4 mg semaglutide cohort 43% versus 33% placebo; p=0.048). In terms of secondary outcomes, there was significant weight loss in the 0.4 mg group (mean % weight loss 13%) compared with the placebo group (1%).

There are some interesting analyses relating to the failure of this trial, some of which mirror the PIVENS study,^17^ which also demonstrated a high level of fibrosis regression in the placebo cohort (31%), findings that are considerably higher than in other similar trials.^44^ A phase III trial of semaglutide in NASH is now being planned.

More recently, the diabetes trial, STEP 2,^46^ demonstrated that patients randomized to semaglutide 2.4 mg (once weekly) resulted in -9.6% mean bodyweight reduction from baseline at week 68 versus -3.4% with placebo, again, highlighting the weight-related improvements associated with semaglutide usage.

### Liraglutide

The LEAN study was a multicentre (four UK centres), phase II, doubleblind RCT assessing the efficacy of subcutaneous liraglutide (1.8 mg daily) compared with placebo.^44^ The trial cohort included those with relative obesity and histological evidence of NASH. The trial design incorporated a randomization minimization of 1:1, stratified by trial centre and diabetes status, whereby 26 patients received liraglutide and 23 received placebo. In those patients who underwent end-of-therapy liver biopsy, 9 of 23 patients (39%) in the treatment group and 2 of 22 patients (9%) in the placebo group achieved resolution of NASH (relative risk [RR] 4.3, 95% confidence interval [CI] 1.9–17.7; p=0.019). In contrast, 9% (2/23) of the treatment group versus 36% (8/22) of the placebo group demonstrated clear progression of fibrosis (RR 0.2, 95% CI 0.1–1.0; p=0.04).

Although the numbers within the trial were small, the encouraging signals provided a tantalizing insight into potential biological potential, which subsequently formed the basis for the semaglutide trial described above. There are no current NASH trials that aim to extend the use of liraglutide.

## Farnesoid X receptor agonists

FXR exists as two entities within humans, FXR-α and FXR-β, although the latter is a pseudogene. As a member of the nuclear receptor family, FXR acts as a ligand-modulated transcription factor, the role of which is to increase or decrease the transcriptional activity of regulated promoters in a coordinated fashion. FXR is a metabolic nuclear receptor and is activated by primary bile acids such as chenodeoxycholic acid, cholic acid and synthetic agonists such as obeticholic acid (OCA). FXR plays a crucial role in regulating cholesterol homeostasis, lipid metabolism, glucose metabolism and the microbiome, all of which likely relate to NASH pathogenesis.^47^

### Obeticholic acid

OCA (or 6-ethylchenodeoxycholic acid) is a bile acid derivative, which is a potent activator of the farnesoid X nuclear receptor, and can reduce liver fat and fibrosis in animal models of NAFLD.^48,49^ The FLINT trial^50^ assessed the efficacy of OCA in patients with biopsy-proven NASH. FLINT categorically assessed response to treatment for non-cirrhotic, non-alcoholic steatohepatitis to assess treatment with OCA given orally (25 mg daily) or placebo for 72 weeks, with patients stratified in a 1:1 allocation ratio by centre or diabetes status.

The primary outcome was improvement in liver histology, defined as decrease in NAS of at least 2 points, with no deterioration of fibrosis staging. The trial included a pre-planned interim analysis of biochemical markers, supporting continuation of the trial. Within the trial, 141 patients were randomized to OCA and 142 received placebo. Results showed that 50 of 110 patients (45%) in the OCA group and 23 of 109 patients (21%) in placebo group who underwent liver biopsy at both baseline and 72 weeks demonstrated improved liver histology (RR 1.9, 95% CI 1.3–2.8; p=0.0002). Unfortunately, there was an unexpected consequence of increased cholesterol and decreased high-density lipoprotein. These sequelae are likely to be due to the fact that functional FXR activation reduces bile acid synthesis by inhibiting the conversion of cholesterol to bile acids. This is a key regulatory step in cholesterol homeostasis. The FLINT trial therefore demonstrated improved histological features of NASH, but long-term safety and utility require further clarification.

Recently, OCA achieved the interim histological endpoint of fibrosis improvement (1,968 patients: 311 placebo, 312 OCA 10 mg, 308 OCA 25 mg) with no worsening of NASH in the phase III REGENERATE study.^51^ The NASH resolution endpoint was unfortunately not achieved (25 [8%] placebo; 35 [11%] OCA 10 mg; 71 [23%] OCA 25 mg). The results from this planned interim analysis identify clinically significant histological improvement that is likely to translate to clinical benefit. This study is ongoing to assess clinical outcomes and is likely to complete in 2025.

Although the REGENERATE study (Clinicaltrial.gov NCT02548351) failed to definitively dispel any lingering concerns around the efficacy of OCA in NASH resolution, there are clear positive signals from what was a well-designed and well-powered study. With regard to concerns around dyslipidaemic features with OCA treatment, the CONTROL study demonstrated good safety, acceptability and low-density lipoprotein cholesterol control with co-administration of atorvastatin with OCA, which should provide confidence in this approach going forward.^52^

## Chemokine receptor antagonists

The chemokine receptor 2 (CCR-2) and receptor 5 (CCR-5) are central orchestrators of leukocyte trafficking in inflammatory processes. Emerging evidence for the role of CCR-2 and CCR-5 receptors in human inflammatory diseases, arteriosclerosis and NASH has led to growing interest in developing CCR-2 and CCR-5 selective antagonists.^53^

### Cenicriviroc

Cenicriviroc is a dual CCR-2 and CCR-5 antagonist under investigation as a putative therapy for NASH.^54^ Recently, year 1 primary analysis of the 2-year CENTAUR study demonstrated that cenicriviroc had an antifibrotic effect without impacting on degree or inducing regression of steatohepatitis.

The CENTAUR study was a randomized, controlled study of adults with NASH, NAS ≥4 and NASH CRN stage 1–3 fibrosis. The innovative study design included a placebo-to-treatment crossover schedule, with patients in arms A and C receiving cenicriviroc 150 mg or placebo, respectively, for 2 years, while patients in arm B received placebo in year 1 and switched to cenicriviroc in year 2. Histological assessment was performed with biopsy at baseline, year 1 and year 2. Of 289 randomized patients, data on 242 entering year 2 were available for analysis. At year 2, 24% of patients who converted to cenicriviroc versus 17% who remained on placebo achieved ≥1-stage fibrosis improvement, with no worsening of NASH (p=0.37). A significant proportion of patients on treatment who achieved fibrosis response at year 1, maintained similar benefit at year 2 (60% arm A versus 30% arm C), including 86% on cenicriviroc who had stage 3 fibrosis at baseline histology. Unfortunately, following 2 years of investigation, an almost identical percentage of patients on cenicriviroc and placebo achieved ≥1-stage fibrosis improvement, again with no worsening of NASH (15% arm A versus 17% arm C). Exploratory endpoints of fibrosis assessment included consistent reductions in levels of N-terminal type 3 collagen pro-peptide and enhanced liver fibrosis scores. Similarly, there were commensurate increases in aspartate aminotransferase-to-platelet ratio index and fibrosis 4 scores observed in apparent non-responders.

The AURORA study (Clinicaltrials.gov NCT03028740) is a dual-phase randomized, double-blind trial of cenicriviroc utilizing surrogate endpoints of fibrosis stage improvement of ≥1 (NASH CRN) and no worsening of steatohepatitis at month 12.^55^ A second phase of the study enrolled additional patients to determine long-term clinical outcomes including histopathological progression to cirrhosis, liver-related clinical outcomes and all-cause mortality. Patients were randomized in a 2:1 ratio to receive cenicriviroc 150 mg once daily or placebo for 40 months. Within these groups, the primary outcome was achieved in 22.3% (95% CI 19.6–25.2) in the cenicriviroc cohort versus 25.5% (95% CI 21.5–29.9) in the placebo arm. None of the additional secondary endpoints were achieved within the reported study outcomes, although not all outcomes have been definitively reported at this point.^55^

Unfortunately, it appears that cenicriviroc is unlikely to form the basis of any further trials in NASH going forward. Although there may as yet be some benefit in relation to cardiovascular sequelae, this is unlikely to be explored in a pure NASH population. While some of the initial data appeared promising, particularly with respect to non-invasive markers of fibrosis, there was poor correlation with histological outcomes in the trial cohort.

## Metabolic enzyme modulators

This is a class of related compounds that target specific aspects of lipogenesis and triglyceride synthesis. It includes acetyl-CoA carboxylase (ACC) inhibitors, stearoyl-CoA desaturase 1 (SCD-1) inhibitors and diacylglycerol acyltransferase 2 (DGAT-2) inhibitors.

### Acetyl-CoA carboxylase inhibitors

Hepatic de novo lipogenesis (DNL) is a potentiator of NAFLD, which may result in an increased triglyceride burden within hepatocytes.^56,57^ A promising approach involves targeting ACC, which catalyses the initial reaction in the DNL pathway whereby acetyl-CoA is converted to malonyl-CoA. Within DNL homeostasis, malonyl-CoA is an essential basic substrate while also functioning as a potent allosteric inhibitor of carnitine palmitoyltransferase 1. Carnitine palmitoyltransferase-1 plays a vital capacitance role in the co-localization of long-chain fatty acyl-CoA across the mitochondrial membrane where it undergoes β-oxidation.^58,59^ The dimerization is catalysed in a stepwise manner, involving both a biotin carboxylase reaction and a carboxyltransferase reaction.^60^

### Diacylglycerol acyltransferase 2 inhibitors

Active inhibition of DGAT-2 induces a reactive downregulation of sterol regulatory element binding protein 1, a potent mediator of glycolysis and inducer of lipogenesis, which suppresses downstream lipogenic modulators and upregulates alternative oxidative processes.^61^ Furthermore, DGAT-2 is central to the esterification of fatty acids with diacylglycerol, producing triglycerides. Previous studies in patients with NAFLD have shown beneficial effects on triglyceride lowering and ameliorating hepatic steatosis.^62,63^

A recent trial examined the possibility of exploiting the potential utility of another novel ACC inhibitor (PF-05221304; 2, 10, 25 and 50 mg) compared with placebo, with an evaluation of relative liver fat fraction at 16 weeks.^64^ A parallel component of the study explored the putative benefit of adding a DGAT-2 inhibitor (PF-06865571 – 300 mg twice daily) as it may additionally offset the potential for hypertriglyceridaemia experienced in ACC inhibitors. Dose-dependent reductions in liver fat were achieved using PF-05221304 and PF-06865571 monotherapy from index MRI to week 6. Placebo-adjusted changes were -44.5% (p<0.0001) and -3.4% (p=0.0007), respectively. Co-administration lowered steatosis by -44.6%, which was relatively equivalent to PF-05221304 monotherapy; however, a greater proportion of patients receiving both therapies achieved >30% or >50% reduction in liver fat burden. While this combination approach provides some tantalizing insights, robust, long-term data with added histological considerations are needed to verify these preliminary data.

### Firsocostat

Firsocostat (GS-0976) is another highly liver-specific, small molecule that binds avidly to the biotin carboxylase regulatory terminal, thereby inhibiting downstream dimerization and consequent ACC activation. Firsocostat is uniquely hepatocyte-specific as it was developed as a substrate for hepatic organic anion-transporting polypeptide transporters.^65^ This results in exclusive hepatic biodistribution of compound delivery, which has favourable therapeutic potency. In a recent open-label trial,^66^ firsocostat was combined with semaglutide +/- cilofexor (FXR agonist), and demonstrated encouraging signals of enhanced liver steatosis resolution (as measured by MRI-PDFF) despite no additional benefit on weight loss (7–10%) versus semaglutide monotherapy. Tolerability overall seemed good, with predominantly gastrointestinal adverse events. It will continue to be evaluated through a number of upcoming trials.

### Stearoyl-CoA desaturase 1 inhibitors

SCD-1 catalyses monounsaturated fatty acids, preferentially stearoyl (C18:0) and palmitoyl (C16:0) CoA using nicotinamide adenine dinucleotide phosphate, cytochrome b5 and associated cytochrome b5 reductase to yield oleic acid (C18:1) and palmitoleic acid (C16:1), respectively.^67,68^

3β-arachidyl amido cholanoic acid (Aramchol; Galmed Pharmaceuticals Ltd, Tel Aviv, Israel) is an oral, liver-specific bile acid derivative that partially antagonizes SCD-1 expression within the hepatic parenchyma, thereby reducing liver triglyceride burden. Animal models have shown histological improvements in both steatohepatitis activity indices and fibrosis.^69^

The ARREST trial was a 52-week, double-blind, placebo-controlled, phase IIb trial that sought to determine the efficacy of Aramchol 400 mg and 600 mg versus placebo in a cohort of 247 patients with NASH.^70^ The primary endpoint was a relative reduction in hepatic triglyceride concentration as measured using magnetic resonance spectroscopy. Secondary endpoints of note included histological assessment and resolution of transaminases. Aramchol 600 mg, unfortunately, failed to reach significance in relation to the primary outcome, thus making all additional analyses exploratory in nature. In determining histological endpoints, NASH resolution, without worsening fibrosis, was noted in 16.7% of patients taking Aramchol 600 mg versus 5% within the control population (odds ratio 4.74). Similarly, resolution of fibrosis by ≥1 stage without worsening steatohepatitis was noted in 29.5% versus 17.5%, respectively.

Again, there appears to be benefit in further exploring the potential additive effects of these agents, particularly in tandem with compounds targeting synergistic pathways. Future trials are likely to employ this strategy to achieve requisite endpoint outcomes.

## Conclusion

In the past decade, there have been significant developments in our understanding of the pathophysiology of NASH, which consequently have led to the development of a number of promising therapeutic interventions. New molecules and pathways are being targeted, while we look to further improve our understanding around metabolomic and genomic contributors to the pathogenesis of NASH. Given the complexity of the underlying pathophysiology and the number of associated conditions, it is likely that a personalized approach may be necessary in order to achieve specific desired endpoints, which may require multiple therapeutic agents.^1^ Current trial reports have highlighted the challenges that exist around histology-based trial endpoints including variability in liver histology interpretation (especially evaluation of ballooning degeneration),^71^ lack of matching patients with particular comorbidities (e.g. diabetes) between phase II and III studies, and strict recording of dietary and exercise during the follow-up period beyond standard treatment timing.^72^ Currently, there are a number of alternative modalities under investigation to determine whether they will prove robust surrogates to traditional histological-based outcomes. Endpoints based on MRI in particular, as a non-invasive modality, may prove effective, particularly for early study designs for drugs that influence hepatic^73,74^ steatotic burden, rather than anti-inflammatory or anti-fibrotic modes of action. However, no biomarker or imaging modality has been fully approved as a replacement for histological assessment to date.

Although there have been enormous developments in the understanding of the pathogenesis of NASH, enabling the development of novel compounds that will hopefully prevent disease progression from NASH to cirrhosis and/or hepatocellular carcinoma, the overriding emphasis should remain one of disease prevention. Population health strategies to reduce the prevalence of obesity and increase the number of individuals engaging in regular exercise are critical to address the rapidly developing challenges of obesity and other related conditions such as diabetes.
